# Translational Advances in Lipid Nanoparticle Drug Delivery Systems for Cancer Therapy: Current Status and Future Horizons

**DOI:** 10.3390/pharmaceutics17101315

**Published:** 2025-10-10

**Authors:** Hari Krishnareddy Rachamala

**Affiliations:** Department of Biochemistry and Molecular Biology, Mayo Clinic College of Medicine and Sciences, Jacksonville, FL 32224, USA; rachamala.hari@mayo.edu

**Keywords:** lipid nanoparticles, liposomes, targeted drug delivery, cancer nanomedicine, theranostics, translational oncology

## Abstract

Lipid nanoparticles/liposomes (LNPs) represent a highly adaptable nanocarrier system that has gained significant traction in oncology for both therapeutic and diagnostic (theranostic) purposes. Their structural flexibility, biocompatibility, and capacity to encapsulate diverse therapeutic agents ranging from chemotherapeutics to nucleic acids and imaging tracers have enabled targeted cancer treatment with improved efficacy and reduced systemic toxicity. This review critically examines liposome-based platforms across a broad spectrum of cancers, including melanoma, lung, colorectal, liver, breast, ovarian, pancreatic, brain tumors, sarcoma, neuroblastoma, and leukemia. It outlines recent advances in ligand-mediated targeting, pH- and temperature-responsive release systems, and multifunctional LNPs capable of delivering combined therapeutic and imaging payloads. Moreover, the review discusses preclinical outcomes, current clinical trial status, and the challenges hindering clinical translation. By integrating recent innovations and emphasizing translational potential, this work highlights the pivotal role of LNPs in advancing precision cancer therapeutics and diagnostics.

## 1. Introduction

Over the past two decades, nanomedicine has emerged as a transformative force in oncology, offering new avenues to overcome the limitations of conventional cancer diagnostics and therapeutics [1]. Traditional chemotherapeutic regimens are often hindered by systemic toxicity, poor bioavailability, multidrug resistance, and a lack of tumor specificity. Nanotechnology provides a versatile platform to address these issues by enabling the design of nano-scaled drug delivery systems that can selectively accumulate in tumor tissues, prolong circulation time, and co-deliver multiple therapeutic agents [2]. Nanomedicine in oncology encompasses a broad spectrum of nanoscale materials including LNPs, polymeric nanoparticles, dendrimers, gold nanoparticles, carbon-based nanostructures, and more recently, LNPs [3]. These platforms can be engineered to navigate the biological complexities of cancer, including the abnormal vasculature of tumors, hypoxic cores, acidic microenvironments, and immunosuppressive niches. One of the most promising aspects of nanomedicine is its potential to enable precision oncology the tailoring of treatment strategies based on individual tumor characteristics [4]. Nanoparticles can be functionalized with ligands that recognize tumor-specific biomarkers, enhancing selective uptake by cancer cells while sparing healthy tissues. Furthermore, nanocarriers facilitate the delivery of novel therapeutic modalities such as RNA interference (RNAi), messenger mRNA (mRNA)-based vaccines, CRISPR-Cas9 gene editors, and immune checkpoint modulators, many of which are challenging to deliver using conventional systems [5]. Recent advancements have also seen the integration of imaging agents into nanocarriers, enabling theranostic approaches that combine therapy and diagnostics in a single platform. This dual functionality paves the way for real-time treatment monitoring and adaptive therapy. Despite these advances, the clinical translation of nanomedicine remains a formidable challenge. Issues related to large-scale manufacturing, regulatory approval, immune compatibility, and inter-patient variability continue to limit the widespread adoption of nanotechnologies in oncology [6]. Nonetheless, the rapid progress in materials science, high-throughput screening, and AI-assisted nanoparticle design is expected to accelerate the development of clinically viable nanomedicines. In this landscape, LNPs have emerged as a frontrunner due to their biocompatibility, flexibility in encapsulating diverse therapeutic cargos, and recent success in mRNA vaccine delivery [7]. Their ability to deliver complex payloads such as small interfering RNA (siRNA), mRNA, and proteins to solid tumors with high precision marks a new era in cancer therapy, underscoring the urgency to explore their full potential in oncological applications.

**Figure 1:**
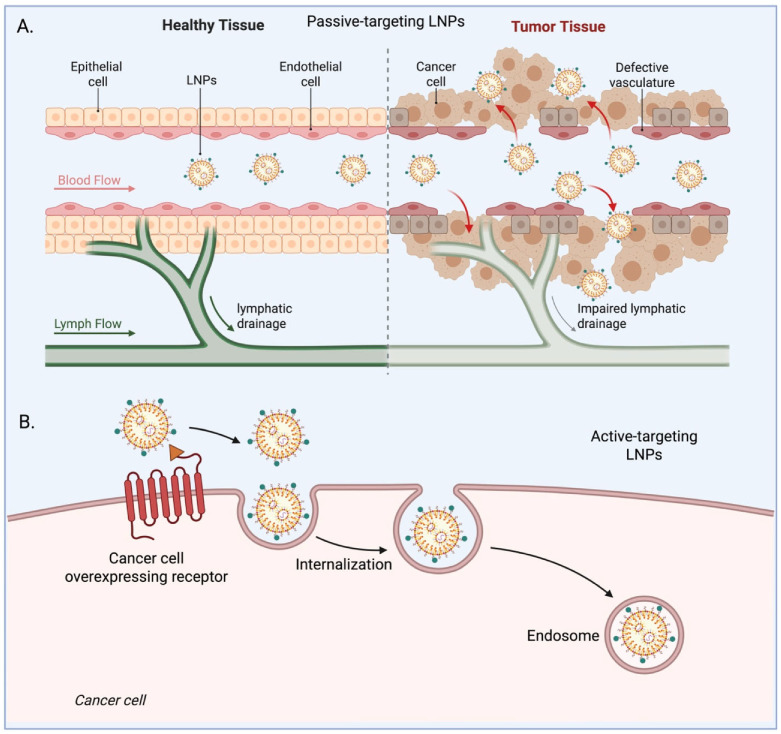
Mechanisms of Passive and Active Targeting of LNPs to Tumor Tissue: (**A**) Schematic representation of passive targeting of LNPs through the EPR effect. In healthy tissue, LNPs circulate through intact vasculature and are efficiently cleared via normal lymphatic drainage. In contrast, tumor tissue exhibits abnormal, leaky vasculature and impaired lymphatic clearance, allowing LNPs to preferentially accumulate in the tumor microenvironment via passive diffusion. (**B**) Illustration of active targeting of LNPs to tumor cells overexpressing specific surface receptors. Ligand-modified LNPs bind to these receptors, undergo receptor-mediated endocytosis, and are internalized into the cancer cells via endosomal pathways, enhancing intracellular delivery of therapeutic cargo.

The development of LNPs represents a significant milestone in the evolution of nanocarrier systems for drug delivery, especially in the context of cancer therapeutics. While early lipid-based systems such as LNPs laid the groundwork for nanoscale delivery platforms, LNPs have rapidly ascended to the forefront of nanomedicine due to their structural versatility, biocompatibility, and exceptional ability to encapsulate and protect labile therapeutic agents [8]. Unlike traditional LNPs, LNPs are typically composed of a mixture of ionizable lipids, phospholipids, cholesterol, and polyethylene glycol (PEG)-lipids, forming a stable, amorphous or non-bilayer structure. This unique architecture allows LNPs to effectively encapsulate both hydrophilic and hydrophobic agents, as well as complex biomolecules like mRNA, siRNA, DNA, and proteins [9]. The modularity of their design also permits precise tuning of particle size, surface charge, and release kinetics, offering an unmatched degree of control over delivery parameters. A defining breakthrough in LNP technology came with the clinical success of mRNA-based COVID-19 vaccines, which validated LNPs as safe and scalable delivery systems for nucleic acid therapeutics. This success has catalyzed a renewed interest in leveraging LNPs for a broad range of biomedical applications, including oncology [10]. LNPs offer an efficient means to overcome key barriers associated with cancer drug delivery such as enzymatic degradation, poor tumor penetration, and off-target toxicity by enhancing payload stability, enabling targeted delivery, and facilitating endosomal escape. In cancer therapy, LNPs have shown great promise in delivering chemotherapeutic drugs, gene-silencing agents (e.g., siRNA), gene-editing tools (e.g., CRISPR/Cas9), and immunomodulators to both primary tumors and metastatic sites [7,8,11,12,13]. Their ability to be surface-modified with targeting ligands (e.g., antibodies, peptides, aptamers) allows for active targeting of tumor-specific markers, improving therapeutic index and minimizing systemic side effects. Moreover, ionizable lipids used in LNP formulations are pH-responsive, facilitating endosomal release of the cargo in the acidic tumor microenvironment or intracellular compartments [14]. Anindita et al. investigated how cholesterol content influences the adjuvant activity of nucleic-acid-free LNPs. Using ovalbumin as a model antigen, they showed that the presence of ionizable lipids is essential but not solely responsible for immunostimulation. Instead, antibody production correlated with the formation of an intermediate intra-particle cholesterol structure, termed the cholesterol-induced phase. When cholesterol levels were too high, crystalline-like domains formed, which diminished adjuvant activity. Cryo-TEM and SAXS analyses confirmed these structural transitions. The study concludes that controlling cholesterol assembly within LNPs can tune their immunostimulatory properties, offering a strategy to optimize LNP-based vaccine adjuvants [15].The hybrid LNPs incorporating inorganic or polymeric components, biomimetic coatings derived from cell membranes to evade immune clearance, and stimuli-responsive systems that release drugs in response to tumor-specific triggers such as redox gradients or enzymes. The application of machine learning algorithms in LNP formulation design is also beginning to reshape the discovery process, enabling predictive modeling of optimal lipid compositions for specific therapeutic payloads and disease targets. As the field progresses, LNPs are increasingly recognized not just as passive delivery vehicles (Figure 1A), but as active modulators of biological responses capable of engaging immune pathways, altering tumor-stromal interactions, and reprogramming the tumor microenvironment [16]. Their emergence marks a paradigm shift in drug delivery science, offering a robust and flexible platform that aligns seamlessly with the demands of next-generation cancer therapeutics.

The integration of nanotechnology into oncology has created new opportunities for targeted and personalized cancer therapy. LNPs stand out among nanocarriers because of their modular design, proven clinical success, and versatility across therapeutic platforms. Initially validated through mRNA vaccine delivery, LNPs are now being advanced for siRNA, CRISPR-based gene editing, and immunotherapy, positioning them as a cornerstone for next-generation cancer therapeutics [17]. Despite the growing body of research, a comprehensive synthesis that critically examines the biomedical applications of LNPs specifically within the context of cancer therapy while integrating recent innovations and anticipating future trends is still needed [18]. This review aims to fill that gap by offering a state-of-the-art overview of how LNPs are being harnessed and evolved to address the complex biological and translational challenges of oncology. The key objectives of this review are as follows such as to elucidate the fundamental principles underlying the design, composition, and functionality of LNPs, with a focus on parameters that influence their behavior in cancer-specific contexts. To dissect the mechanisms by which LNPs achieve tumor-specific delivery, including passive and active targeting strategies (Figure 1B), and stimuli-responsive designs that exploit the tumor microenvironment. To highlight the diverse therapeutic payloads ranging from small-molecule drugs to nucleic acids and immunomodulators that can be efficiently delivered using LNPs, along with the synergistic effects seen in combination therapies. To provide a detailed account of recent preclinical breakthroughs and clinical advancements, including LNPs in ongoing oncology trials and FDA-approved or investigational formulations. To critically evaluate the current limitations such as immunogenicity, off-target effects, scale-up challenges, and regulatory hurdles and propose strategies to overcome them. To explore cutting-edge innovations and future directions, including smart and biomimetic LNPs, AI-assisted formulation design, and applications in precision oncology, theranostic, and tumor microenvironment reprogramming. By consolidating and analyzing the rapidly evolving landscape of LNP-based cancer therapy, this review aspires to serve as a comprehensive resource for researchers, clinicians, and developers guiding the rational design and accelerated translation of LNP platforms from bench to bedside.

LNP mediated delivery of therapeutics: LNPs are versatile delivery vehicles designed to transport therapeutic agents, including small molecules, nucleic acids (mRNA, siRNA, DNA), and proteins, to target cells. Their structure typically consists of an ionizable lipid core or phospholipids, helper lipids, cholesterol, and a polyethylene glycol (PEG)-lipid component, allowing them to encapsulate cargo efficiently, protect it from degradation, and facilitate cellular uptake [19]. Upon administration, LNPs circulate in the bloodstream and, depending on their design, accumulate in target tissues through passive mechanisms (such as the enhanced permeability and retention effect) or active ligand-mediated targeting. Once at the target site, LNPs are internalized primarily via endocytosis. Within the acidic endosomal environment, ionizable lipids become protonated, destabilizing the endosomal membrane and promoting the release of therapeutic cargo into the cytoplasm [20]. This controlled delivery improves the bioavailability, efficacy, and safety profiles of therapeutics by enhancing their stability, enabling site-specific action, and minimizing systemic side effects. LNP-mediated delivery has been successfully applied in vaccines (e.g., COVID-19 mRNA vaccines), gene therapies, cancer therapeutics, and the treatment of genetic disorders [21,22].

Delivery of chemotherapeutic drugs: Lipid nanoparticles (LNPs) provide a versatile and effective platform for delivering chemotherapeutic agents, with the primary goal of enhancing their therapeutic index while minimizing systemic toxicity. Traditional chemotherapy is often limited by poor solubility, rapid systemic clearance, non-specific biodistribution, and dose-limiting side effects. LNPs overcome these barriers by encapsulating drugs within a stabilizing lipid matrix, thereby enhancing drug solubility, prolonging circulation time, and facilitating tumor-specific delivery [23]. Several studies have demonstrated the potential of LNPs to improve cancer therapy. Rachamala et al. developed a tumor-targeted liposomal formulation (EY-L) co-encapsulating everolimus and YM155 to enhance the radiosensitivity of renal cell carcinoma (RCC). This dual-drug system significantly suppressed tumor growth and amplified the efficacy of radiation therapy in both in vitro and in vivo RCC models by impairing DNA damage repair and inducing mitotic catastrophe. Compared to single-agent formulations, EY-L exhibited superior antiproliferative effects, modulated the tumor immune microenvironment, and achieved synergistic inhibition of mTOR and survivin signaling pathways offering a compelling strategy to overcome radio resistance in RCC and potentially other malignancies [11]. Similarly, Gabriel et al. reported that liposomal phenylephrine nanoparticles (LPE-NPs) enhanced the efficacy of intratumoral chemotherapy in a preclinical melanoma model. The LPE-NPs improved drug retention, modulated tumor vasculature, and increased local cytotoxicity, resulting in superior tumor control and reduced systemic toxicity [24]. In another study, Rachamala et al. demonstrated that liposomal encapsulation of thymoquinone significantly improved its pharmacokinetic profile, promoted tumor-specific accumulation, and boosted antitumor efficacy while minimizing systemic adverse effects. Following intravenous administration, LNPs preferentially accumulate in tumor tissues via the enhanced permeability and retention (EPR) effect. This passive targeting can be further refined through surface functionalization with tumor-specific ligands such as antibodies, peptides, or aptamers that enable selective binding to overexpressed tumor markers and enhance cellular uptake [25]. Once internalized, LNPs promote endosomal escape and facilitate cytoplasmic release of their payload, where chemotherapeutic agents exert their cytotoxic effects. Furthermore, LNPs can be engineered to respond to tumor-specific stimuli such as acidic pH, elevated glutathione levels, or tumor-associated enzymatic activity enabling controlled and localized drug release [26]. This stimulus-responsive behavior not only enhances intratumoral drug activation but also minimizes off-target effects and helps circumvent multidrug resistance by evading efflux transporters and supporting combination therapy strategies.

LNP mediated delivery of RNA based therapeutics: LNPs are the leading delivery systems for RNA-based therapeutics, including siRNA, mRNA, and CRISPR/Cas genome editing components [27]. RNA molecules are inherently unstable and susceptible to rapid degradation by nucleases in biological fluids. LNPs protect these sensitive cargos, enhance their circulation time, promote cellular uptake, and enable efficient intracellular release. LNPs encapsulate siRNA molecules to enable gene silencing by RNA interference (RNAi). After endocytosis, siRNA is released into the cytoplasm, where it binds the RNA-induced silencing complex (RISC) and leads to the degradation of target mRNA, thus downregulating pathogenic genes involved in cancer, fibrosis, and genetic diseases [28]. Rachamala et al. developed a novel lipid-based nanocarrier designed for dual targeting of Syndecan-1 and Glucose Transporter-1 (GLUT1), demonstrating its ability to significantly enhance therapeutic efficacy and overcome chemoresistance in pancreatic ductal adenocarcinoma (PDAC). This dual-targeted formulation facilitated selective tumor accumulation, enhanced intracellular drug delivery, and elicited potent cytotoxic effects in both in vitro and in vivo models. Mechanistically, the strategy disrupted critical survival pathways and impaired the metabolic flexibility of PDAC cells, leading to increased apoptosis and substantial tumor regression. These results highlight the promise of dual-receptor targeted lipid nanocarriers as a therapeutic strategy for treatment-refractory PDAC and support their further development toward clinical application [8]. Rachamala et al. demonstrated that lipid composition critically influences the therapeutic efficacy and genotoxicity of anti-tumor nano-lipoplexes. Both unsaturated and saturated lipid-based formulations exhibited strong antitumor activity; however, they elicited distinct genotoxic responses in vivo. Lipoplexes containing unsaturated lipids showed a more favorable safety profile, with minimal DNA damage, whereas those formulated with saturated lipids induced higher levels of genotoxic stress. These results highlight the importance of lipid saturation in modulating both therapeutic performance and off-target effects. The study underscores the necessity of rational lipid selection in designing safe and effective gene delivery systems for cancer therapy, with future investigations warranted to optimize lipid composition for improved clinical outcomes [13]. LNPs have been widely used to deliver therapeutic mRNA, most notably in mRNA vaccines (e.g., COVID-19 vaccines). After cellular uptake and endosomal escape, the mRNA is translated by the host’s ribosomes into therapeutic proteins, such as antigens for immune activation, tumor suppressors for cancer therapy, or enzymes for metabolic disorders [29]. This study demonstrates that the SMART-lipid nanoparticle (SMART-LNP) platform enables robust delivery of mRNA vaccines, eliciting potent and cross-reactive humoral immune responses against multiple SARS-CoV-2 Omicron sub-variants. The optimized SMART-LNPs enhanced mRNA stability, facilitated efficient antigen expression, and promoted strong neutralizing antibody responses with broad variant coverage. These findings underscore the potential of SMART-LNP-based mRNA vaccines to provide durable and adaptable protection against evolving viral strains, supporting their continued development as a next-generation platform for pandemic preparedness and variant-specific immunization strategies [30]. LNPs can co-deliver mRNA encoding Cas nucleases and guide RNA (gRNA) or directly deliver Cas9/gRNA ribonucleoprotein complexes. Once inside the cell, the CRISPR/Cas system enables precise genome editing by introducing site-specific double-stranded breaks, allowing for gene correction, disruption, or insertion [31]. This study presents a novel liposomal system that significantly enhances the production efficiency of SARS-CoV-2 pseudo virion using a lentiviral backbone. The optimized formulation improved transfection efficiency, increased pseudo virus yield, and preserved functional spike protein incorporation, enabling reliable and scalable pseudo virus generation for downstream applications. This platform offers a valuable tool for high-throughput screening of neutralizing antibodies, vaccine candidates, and antiviral therapeutics. The findings highlight the potential of liposome-assisted delivery systems in advancing pseudo virus-based virological research and accelerating COVID-19 countermeasure development [32]. LNP formulations typically use ionizable lipids that remain neutral during circulation but become positively charged in the acidic endosome, facilitating endosomal disruption and cytoplasmic release. Additionally, surface modifications can enhance tissue-specific delivery, reduce off-target effects, and improve therapeutic efficiency [32]. LNPs encapsulate mRNA encoding tumor-associated antigens (TAAs) or pathogen-derived antigens. Upon delivery to antigen-presenting cells (APCs) such as dendritic cells, the mRNA is translated into proteins that trigger a robust adaptive immune response, stimulating cytotoxic T cells and antibody production [33]. Overall, LNP-mediated delivery of RNA therapeutics has transformed modern medicine, enabling new treatment paradigms for infectious diseases, cancer, genetic disorders, and regenerative medicine.

LNP mediated delivery of immunotherapeutic cargo: LNPs have emerged as highly effective carriers for delivering immunotherapeutic agents aimed at modulating the immune system for cancer therapy, infectious diseases, and autoimmune disorders. Their ability to encapsulate and protect a wide range of biomolecules, combined with targeted delivery and controlled release capabilities, makes them ideal for immunotherapy applications [34]. A 2025 study reported a lyophilizable lipid nanoparticle (LNP) vaccine encapsulating an E7 peptide antigen along with Mn^2+^ as a STING agonist for post-surgical adjuvant immunotherapy. The formulation elicited strong CD8^+^ T-cell responses, prevented tumor recurrence, and generated long-lasting immune memory in murine cervical cancer models. Importantly, when combined with anti-PD-1 checkpoint blockade, the LNP vaccine demonstrated synergistic tumor regression, highlighting its translational potential for durable cancer immunotherapy [35]. Hamouda et al. uses LNPs to deliver a mixture of mRNAs encoding immunostimulatory molecules (IL-21, IL-7, 4-1BBL) via intratumoral injection. It shows increased tumor-infiltrating CD8^+^ T cells, enhanced cytokine production (IFN-γ, granzyme B), tumor rejection, and immunological memory in mice [36]. Chai et al. compare LNP formulations for delivering DNA constructs encoding antigens like spike protein, PD-L1, p53 mutants, or monoclonal antibodies. It reports strong antigen-specific antibody and T-cell responses; in animal models, some of these biologics inhibited tumor growth or metastasis [37]. LNPs can deliver cytokines (e.g., IL-2, IL-12), immune checkpoint inhibitors (e.g., anti-PD-1 or anti-CTLA-4 peptides/mRNA), or adjuvants (e.g., TLR agonists) directly to immune cells or the tumor microenvironment, enhancing the anti-tumor immune response while minimizing systemic toxicity. LNPs can deliver RNA molecules that silence immune-suppressive genes in tumors (e.g., TGF-β, IDO) or in regulatory immune cells, reprogramming the tumor microenvironment to favor immune activation [38]. LNPs enable vivo gene editing of immune cells, such as engineering T cells to enhance their cancer-fighting capabilities or knock out inhibitory genes that limit immune responses. Upon systemic administration, LNPs preferentially accumulate in lymphoid tissues or tumors, depending on their design, and promote efficient uptake by key immune cells. Stimuli-responsive modifications and ligand targeting can further enhance selective delivery to specific immune subsets, improving the therapeutic index [39]. Overall, LNP-mediated delivery of immunotherapeutic cargos represents a next-generation approach to precisely and effectively harness the immune system for disease treatment.

Next-Generation LNPs for precision therapeutics: Recent advancements in LNP technology have paved the way for next-generation delivery platforms with enhanced precision, safety, and therapeutic efficacy [10]. These innovations are driven by the integration of advanced ionizable lipids, biodegradable linkers, and organ-targeting components, enabling the efficient and selective delivery of a wide range of payloads including mRNA, siRNA, and CRISPR-Cas systems for applications in cancer therapy, vaccines, and gene editing. Biomimetic and cell membrane-coated LNPs have emerged as powerful strategies to improve immune evasion, circulation half-life, and tissue-specific targeting. By incorporating natural components such as cancer cell membranes or leukocyte-derived surfaces, these carriers mimic biological interfaces, allowing for enhanced biocompatibility and reduced clearance by the mononuclear phagocyte system [40]. This approach significantly improves delivery to tumors or immune organs, supporting applications in personalized immunotherapy and beyond. At the core of LNPs, ionizable lipids play a critical role in facilitating intracellular delivery. These lipids remain neutral in physiological conditions, minimizing systemic toxicity, but acquire a positive charge in acidic endosomal compartments to promote endosomal escape and cytosolic release of the nucleic acid cargo [23]. Cutting-edge formulations now employ next-generation ionizable lipids such as SM-102, ALC-0315, and novel biodegradable analogs for improved delivery efficiency and tolerability. In parallel, LNP surfaces are being functionalized with targeting moieties including GalNAc (for hepatocytes), RGD peptides (for tumor vasculature), antibodies, and aptamers to achieve receptor-mediated uptake and tissue-specific localization. Dual and multiplex targeting strategies are being increasingly employed to navigate complex biological barriers, especially within solid tumors and lymphoid tissues [8]. Artificial intelligence (AI) is revolutionizing the LNP development pipeline by enabling high-throughput screening, rational design, and predictive modeling. Machine learning algorithms analyze extensive lipid libraries and delivery outcomes to identify optimal lipid combinations, helper components, and formulation parameters [23]. AI models also simulate biological interactions and predict organ-specific biodistribution, thereby accelerating the design of LNPs with optimal pharmacokinetics, immunogenicity, and therapeutic index. Recent efforts in generative AI further support the de novo design of synthetic lipids with tunable physicochemical properties tailored for specific disease contexts. In the field of personalized oncology, LNPs are transforming treatment paradigms by enabling the delivery of individualized therapies [41]. LNP-encapsulated mRNA vaccines encoding patient-specific neoantigens can activate potent T cell responses against tumors, while siRNA or CRISPR-based approaches allow for the silencing or correction of oncogenic drivers. By incorporating tumor-targeting ligands or autologous membrane coatings, these personalized LNPs achieve high tumor selectivity and minimal off-target effects. AI-guided formulation further refines these nanoparticles to match the molecular and immunological profiles of individual tumors, supporting the development of adaptive, precision-guided cancer treatments [42]. 

AI-Driven advances in the rational design and manufacturing of LNPs: Recent advances in AI have significantly accelerated the rational design and optimization of LNPs, offering new opportunities to improve their therapeutic performance and translational potential (Table 1). One important contribution comes from the work of Wei Wang et al., who developed a deep learning-based virtual screening strategy that evaluated millions of ionizable lipid candidates and successfully identified several molecules with superior delivery efficiency compared to existing benchmark lipids [43]. Similarly, the AGILE platform integrated combinatorial chemistry with machine learning to tailor ionizable lipids toward distinct cellular contexts, thereby enabling rapid and cell type-specific lipid discovery [44]. Beyond lipid chemistry, more advanced architectures such as TransLNP employ transformer-based modeling to integrate both sequential molecular features and three-dimensional spatial representations, allowing for accurate prediction of transfection performance while also addressing common challenges such as data imbalance and limited experimental datasets [45].

AI has also been increasingly applied to improve the manufacturing side of LNPs. Machine learning frameworks, including XGBoost and Bayesian optimization approaches, have been used to refine microfluidic parameters and lipid ratios, enabling better control over particle size, encapsulation efficiency, and reproducibility during scale-up. Such process-level innovations are particularly critical for ensuring consistency in clinical-grade formulations [46]. Together, these advances illustrate how AI not only accelerates the discovery of new ionizable lipids but also enhances formulation predictability and quality control, supporting the design of safer and more effective LNPs. Importantly, the integration of AI into LNP development is expected to expand their applications in precision oncology, tumor microenvironment reprogramming, and theranostics, where highly tailored and reliable delivery systems are required to achieve durable clinical benefit.

Preclinical and clinical advancements: Preclinical studies show that LNPs can deliver mRNA, siRNA, and CRISPR tools with high precision to tumors and immune cells using targeting ligands and biomimetic coatings. Cancer cell membrane-coated LNPs enhance tumor homing and immune activation. Personalized mRNA cancer vaccines, like BioNTech’s BNT122 and Moderna’s mRNA-4157, are in clinical trials for melanoma and solid tumors. CRISPR-LNP therapies, such as Intellia’s NTLA-2001, have shown promising gene editing in vivo. These platforms demonstrate safe, systemic delivery and durable therapeutic effects. Together, they are advancing precision oncology and gene therapy with high specificity and reduced toxicity.

Key preclinical successes of LNPs: Preclinical successes of LNPs have paved the way for transformative therapies. mRNA cancer vaccines, delivered via LNPs, have demonstrated robust tumor-specific immune responses in animal models, showing significant tumor regression [47]. CRISPR-Cas9 LNPs have successfully mediated gene editing in vivo, such as targeting PCSK9 in the liver to lower cholesterol, with high precision and minimal off-target effects. siRNA-LNPs targeting oncogenes like KRAS and MYC have shown effective tumor growth inhibition in models of pancreatic and colorectal cancers [48]. Organ-targeting LNPs (SORT) have enabled selective delivery to tissues beyond the liver, including the lungs, spleen, and lymph nodes. Biomimetic LNPs, such as those coated with macrophage or cancer cell membranes, enhanced tumor targeting and immune activation. LNPs for CAR-T cell engineering have facilitated non-viral, in vivo T cell reprogramming, creating possibilities for off-the-shelf immunotherapies [49]. These preclinical advancements highlight LNPs as a versatile tool for precision oncology and gene therapy.

Clinical trials landscape (2020–2025) of LNPs: From 2020 to 2025, LNPs have made major strides in clinical trials, especially in mRNA vaccines (Table 2). Moderna’s mRNA-1273 and Pfizer/BioNTech’s BNT162b2 demonstrated LNP-based mRNA vaccines’ effectiveness against COVID-19, marking a breakthrough in vaccine delivery [50]. Personalized cancer vaccines, like BNT122 and mRNA-4157, have advanced, using LNPs to deliver neoantigens for targeted immune responses. In gene editing, trials such as NTLA-2001 by Intellia are exploring LNPs for delivering CRISPR-Cas9 gene therapies for genetic diseases [51]. RNAi therapies like Patisiran (ONPATTRO) have expanded, treating rare genetic diseases through LNP delivery [52]. Cancer immunotherapies and CAR-T cell therapies are being enhanced with LNPs, delivering immunomodulatory agents and mRNA. LNPs are also being tested for autoimmune diseases and gene therapies, showing promise in targeting specific tissues [53]. These developments highlight LNPs as key enablers of precision medicine and next-generation therapeutics. The landscape reflects a shift towards safer, more targeted drug delivery systems.

Regulatory challenges and safety profiles of LNPs: Regulatory challenges for LNPs primarily involve ensuring consistent quality and reproducibility in large-scale manufacturing, as well as meeting stringent safety standards. Long-term toxicity is a key concern, as LNPs can accumulate in tissues such as the liver or lungs, potentially causing adverse effects [54]. Regulatory agencies like the FDA and EMA require comprehensive preclinical data, including biodistribution, pharmacokinetics, and immunogenicity studies. Biodegradable ionizable lipids are a focus to minimizing long-term toxicity, but accumulation of LNPs in tissues remains a challenge. The immune response to LNPs, including activation of the complement system or unwanted inflammation, must be thoroughly evaluated [55]. Despite these concerns, LNPs have shown a favorable safety profile in recent clinical trials, especially in vaccines. Clinical data supports the efficacy and safety of LNPs for mRNA vaccines, with minimal side effects in most cases. As more LNP-based therapeutics enter the market, regulatory bodies are refining guidelines on batch consistency, purity, and patient monitoring. Personalized formulations pose additional regulatory hurdles, as each design may require tailored evaluations [56]. Overall, while LNPs show great promise, regulatory frameworks must evolve to address these complex challenges.

Challenges and limitations of LNPs: LNPs face several challenges and limitations in drug delivery, one major hurdle is targeting specificity, as LNPs often have broad tissue distribution, which can lead to off-target effects. Endosomal escape remains a critical challenge, as LNPs must efficiently release their payload inside cells without being degraded in lysosomes [57]. Stability during storage and transportation, especially for mRNA and other nucleic acids, require advanced formulations to prevent degradation. Immunogenicity is another concern, as LNPs can trigger immune responses, causing inflammation or hypersensitivity reactions [58]. The manufacturing complexity of LNPs, including the need for high-quality raw materials and controlled conditions, limits scalability. Biocompatibility and long-term safety remain a focus, as LNP accumulation in organs like the liver can pose risks. Additionally, cost and regulatory approval timelines for new LNP-based therapies can be prohibitive [59]. Despite these limitations, continued advancements in lipid composition, targeting strategies, and manufacturing techniques are helping overcome many of these challenges. LNPs are also constrained by delivery barriers to certain tissues like the brain or tumors. As research progresses, these limitations are being addressed for more effective and precise drug delivery systems.

**Figure 2:**
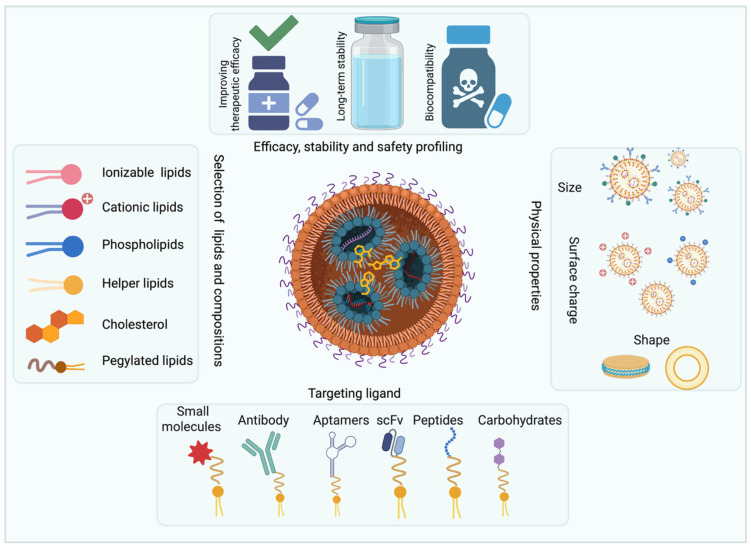
Key Design Parameters Influencing the Performance of LNPs: This schematic illustrates the critical factors affecting the efficacy, stability, and safety of lipid nanoparticles for drug and gene delivery. The core components include various lipid types such as ionizable lipids, cationic lipids, phospholipids, helper lipids, cholesterol, and PEGylated lipids, each contributing to nanoparticle structure and function. Physical properties including particle size, surface charge, and shape play essential roles in biodistribution, cellular uptake, and endosomal escape. Functionalization with targeting ligands (e.g., small molecules, antibodies, aptamers, scFvs, peptides, or carbohydrates) enables receptor-mediated targeting for improved therapeutic precision. Together, these parameters determine the overall biocompatibility, long-term stability, and therapeutic efficacy of LNP formulations.

Stability, scalability, and manufacturing difficulties of LNPs: The stability of LNPs is a major concern, particularly for mRNA and RNA-based therapies, as they require careful formulation to prevent degradation during storage and transport. Temperature sensitivity is a key issue, especially for vaccines, as most LNPs need to be stored at ultra-low temperatures. Scalability remains a challenge, as the complex formulation process for LNPs is difficult to replicate on a large scale while maintaining consistency and quality [60]. Manufacturing difficulties include precise control of lipid composition, particle size, and the encapsulation efficiency of nucleic acids, all of which are critical for performance (Figure 2). High-quality raw materials and specialized equipment are required for production, making it costly. Batch-to-batch variability can occur, leading to inconsistent therapeutic outcomes, which raises concerns for regulatory approval [61]. Additionally, production costs for LNP-based therapies are high, limiting accessibility and widespread use. Large-scale production needs to ensure scalability without compromising purity or efficiency. Advancements in microfluidics and automated production systems are helping address these challenges, but they are still being refined. Overcoming these manufacturing hurdles is essential for making LNPs a mainstream solution in drug delivery. 

Immune system interactions and off-target effects of LNPs: LNPs can interact with the immune system, sometimes triggering unintended immune responses. The complement system can be activated by LNPs, leading to inflammation, allergic reactions, or cytokine storms in some cases. Phagocytosis by macrophages and dendritic cells may clear LNPs from circulation prematurely, reducing their therapeutic efficacy. The lipid composition of LNPs plays a key role in immune activation, with some ionizable lipids and PEGylated lipids potentially inducing immune responses [62]. Off-target effects occur when LNPs are distributed to unintended organs, such as the liver or lungs, causing tissue damage or unwanted immune activation. This can be particularly concerning with cancer therapies, where precise targeting to tumor cells is crucial [63]. Furthermore, antibody formation against LNP components, like PEG, may result in reduced efficacy upon repeated dosing. Biomimetic coatings, such as cancer cell membrane coatings, aim to improve targeting and minimize immune activation [64]. Researchers are working on modulating LNP surface charge and coating strategies to minimize these immune system interactions. Advances in personalized LNP formulations aim to reduce these off-target effects for better therapeutic outcomes.

Biodistribution and pharmacokinetics challenges and limitations of LNPs: Biodistribution and pharmacokinetics of LNPs present significant limitations and challenges in drug delivery. One key issue is non-specific accumulation of LNPs in unintended tissues, particularly the liver, spleen, and lungs, which can lead to off-target effects or toxicity [65]. Achieving targeted delivery to specific tissues, such as tumors or immune cells, remains difficult due to the broad distribution profile of LNPs in vivo [66]. Rapid clearance by the reticuloendothelial system (RES), particularly macrophages, can reduce the effectiveness of LNPs, limiting their half-life in circulation and reducing therapeutic exposure [67]. Additionally, poor penetration into certain tissues like the brain or solid tumors is a major challenge for therapies targeting these areas. The pharmacokinetics of LNPs, including their uptake, biodistribution, and elimination, can vary widely based on the lipid composition, size, surface charge, and encapsulated payload. Biodistribution studies must consider both systemic and local delivery, and variations in individual patients may affect the predictability of LNP behavior. Overcoming these challenges involves designing targeted LNPs with surface modifications, optimizing particle size for efficient tissue penetration, and using strategies like PEGylation or biomimetic coatings to prolong circulation [68]. Advances in AI-driven formulation and microfluidics aim to address these biodistribution challenges by designing more predictable and controlled LNP delivery systems.

Imaging and therapeutic applications of LNPs in various cancers: LNPs have emerged as versatile nanocarriers for both imaging and therapy in oncology due to their biocompatibility, tunable surface properties, and capacity for drug encapsulation. In cancer imaging, LNPs can be functionalized with contrast agents or fluorescent dyes for enhanced tumor visualization using MRI, PET, or optical imaging [69]. For therapy, LNPs enable targeted delivery of chemotherapeutics, reducing systemic toxicity and enhancing drug accumulation in tumors via the enhanced permeability and retention (EPR) effect [70]. Thermosensitive and pH-responsive LNPs allow controlled drug release in the tumor microenvironment. Additionally, ligand-targeted LNPs can selectively bind to cancer-specific markers, improving treatment specificity [71]. Their dual diagnostic and therapeutic (theranostic) capabilities make LNPs promising tools in personalized cancer management.

## 2. LNP-Based Drug Delivery Systems for the Treatment of Melanoma

According to the NIH/NCI Surveillance, Epidemiology, and End Results (SEER) Program, an estimated 104,960 new cases of melanoma of the skin will be diagnosed in 2025, accounting for 5.1% of all new cancer cases. Despite a high 5-year relative survival rate of 94.7%, melanoma is projected to cause 8430 deaths, representing 1.4% of all cancer-related deaths [72]. Genetic mutations, particularly in the BRAF gene and overexpression of AKT3, result in the dysregulation of MAPK and PI3K/AKT signaling pathways, promoting tumor cell proliferation, angiogenesis, and resistance to apoptosis. LNPs based drug delivery systems have emerged as promising tools to address these oncogenic drivers by enabling precise and efficient delivery of therapeutic payloads, including small molecules, mRNA, siRNA, and immunomodulatory agents [73].

Recent advances have demonstrated that LNPs encapsulating doxorubicin and cyclophosphamide exhibit potent antitumor effects in pulmonary metastatic melanoma models by improving drug solubility, biodistribution, and tumor accumulation. Co-delivery strategies, such as LNPs loaded with ceramide and the RAF kinase inhibitor sorafenib, have shown synergistic cytotoxicity in BRAF-mutant melanoma through concurrent targeting of survival and apoptotic pathways [74]. In the realm of cancer immunotherapy, LNPs formulated with melanoma-associated antigen MART-1 mRNA has been shown to stimulate antigen-specific CD8^+^ and CD4^+^ T-cell responses, leading to effective tumor rejection and memory formation in preclinical models. Similarly, BAX mRNA delivered via cationic LNPs composed of DOTAP and DOPE has induced apoptosis in malignant melanoma by enhancing caspase activity and promoting TUNEL-positive cell death [75]. Gene-silencing approaches using LNPs encapsulating siRNA against AKT3 and BRAF have demonstrated significant tumor inhibition when combined with ultrasound-assisted topical delivery, improving transdermal penetration and intracellular uptake. Surface modification of LNPs with targeting ligands such as folic acid, transferrin, and GD2 antibodies has enabled receptor-mediated endocytosis and selective delivery to melanoma cells, enhancing therapeutic precision and reducing off-target toxicity [59]. Theranostic applications have also gained traction, with the development of LNPs conjugated to radiolabeled agents (e.g., bismuth-213) or magnetic nanoparticles for simultaneous imaging and therapy. These platforms allow for real-time tracking of biodistribution and therapeutic efficacy via SPECT, PET, or magnetic resonance imaging. A notable advancement includes the design of siRNA-loaded neutral DOPC-LNPs targeting protease-activated receptor-1 (PAR-1), a thrombin receptor implicated in melanoma metastasis and angiogenesis. These LNPs demonstrated marked suppression of tumor growth and downregulation of VEGF, IL-8, and MMP-2 in murine models [76]. Despite these encouraging preclinical results, no LNP-based formulation for melanoma has yet reached clinical approval. Challenges such as tumor heterogeneity, immune evasion, and scalable manufacturing must be addressed to advance these platforms toward clinical translation. However, ongoing innovations in LNP chemistry, targeting strategies, and immune activation hold significant promises for transforming melanoma therapy.

## 3. Liposome-Based Drug Delivery Systems for the Treatment of Lung Cancer

In 2025, an estimated 226,650 new cases of lung and bronchus cancer will be diagnosed in the U.S., accounting for 11.1% of all new cancer cases. It remains the leading cause of cancer death, with 124,730 projected deaths (20.2% of all cancer deaths). The overall 5-year relative survival rate is 28.1% (2015–2021) [77]. Numerous risk factors contribute to the pathogenesis of lung cancer, including tobacco exposure, environmental pollutants (e.g., arsenic, radon, and industrial particulates), chronic inflammation, and genetic predispositions. At the molecular level, mutations in tumor suppressor genes such as TP53 and RB1, along with aberrant activation of oncogenic kinases including EGFR, MET, and PIK3CA, drive tumor progression and resistance to conventional therapies [78].

Recent studies have demonstrated the efficacy of liposomes conjugated with targeting ligands to improve selective drug accumulation in lung tumors. For instance, GE11 peptide-modified liposomes encapsulating doxorubicin demonstrated enhanced binding affinity to EGFR-overexpressing NSCLC cells, leading to increased cellular uptake via clathrin-mediated endocytosis and superior cytotoxic effects compared to unmodified formulations [79]. These EGFR-targeted liposomes also facilitated near-infrared fluorescence imaging, aiding real-time tumor visualization and biodistribution assessment. To enhance intratumorally penetration and overcome hypoxic barriers, Lin et al. developed triptolide-loaded liposomes co-modified with carbonic anhydrase IX (CA IX) antibodies and CPP33 peptides. These dual-ligand liposomes exhibited superior penetration into 3D NSCLC spheroids and demonstrated significant induction of apoptosis in vitro and in vivo [80]. Multifunctional liposomes co-encapsulating honokiol and epirubicin within a lipid bilayer and decorated with octreotide (OCT) a somatostatin receptor-targeting peptide—have shown promise in suppressing metastasis and vasculogenic mimicry. Mechanistically, these liposomes downregulated PI3K, MMP-2, MMP-9, and VE-cadherin expression, while promoting caspase-3 activation and apoptosis in NSCLC models [81]. Gene-silencing strategies have also been explored. Liposomes coated with guanidinylated cationic amphiphiles encapsulating synthetic CDC20 siRNA effectively suppressed tumor growth in B16F10 lung metastatic models following systemic administration [82]. These formulations achieved enhanced tumor localization and reduced off-target accumulation due to the electrostatic interactions between the cationic surface and negatively charged tumor vasculature. Inhalable liposomal formulations are an emerging platform aimed at maximizing pulmonary drug exposure while minimizing systemic side effects. Phase I clinical studies have assessed sustained-release liposomal cisplatin and interleukin-2 (SLIT-IL-2) aerosols, showing favorable safety and tolerability profiles [83]. Radiation-guided delivery systems have also been developed. Liposomes functionalized Moreover, Makale et al. engineered doxorubicin-loaded PEGylated liposomes with a dextran core for MRI-based imaging of Lewis lung carcinoma, demonstrating dual therapeutic and diagnostic (theranostic) utility [84]. with the HVGGSSV peptide a tumor vasculature-homing sequence enabled selective accumulation in irradiated tissues, enhancing doxorubicin delivery post-radiotherapy. Additionally, liposomes labeled with near-infrared dyes (e.g., Alexa Fluor 750) have been employed to study biodistribution and real-time tracking of nanoparticle behavior using non-invasive imaging platforms [85].

Despite the extensive preclinical success and early clinical investigations, no liposomal formulation for lung cancer has yet reached market approval. Continued research is focused on refining targeting specificity, enhancing pulmonary delivery, and integrating multifunctional capabilities for simultaneous therapy and diagnostics. The convergence of liposomal engineering with advanced imaging, personalized medicine, and immunotherapy holds significant promise for improving lung cancer outcomes.

## 4. Liposome-Based Drug Delivery Systems for the Treatment of Colorectal Cancer (CRC)

According to the NIH/NCI Surveillance, Epidemiology, and End Results (SEER) Program Cancer Stat Facts on colorectal cancer, an estimated 154,270 new cases are expected in 2025, comprising 7.6% of all new cancer diagnoses. Colorectal cancer is projected to cause 52,900 deaths, accounting for 8.6% of all cancer-related deaths. The 5-year relative survival rate from 2015 to 2021 is 65.4% [86]. Despite advancements in early detection and surgical interventions, therapeutic outcomes for advanced CRC remain suboptimal due to metastasis, drug resistance, and systemic toxicity associated with conventional chemotherapeutics. Nanotechnology-based strategies, particularly liposome-based drug delivery systems, have emerged as a promising approach to enhance the therapeutic index, reduce off-target effects, and improve tumor-selective delivery in CRC management [87].

Recent preclinical studies have demonstrated significant advances in CRC therapy using liposomal formulations. For example, liposomes radiolabeled with rhenium-188 (^188^Re) showed potent antitumor efficacy in C26 murine models by inhibiting tumor growth and extending median survival beyond that achieved by 5-fluorouracil [88]. Similarly, hybrid liposomes incorporating L-α-dimyristoylphosphatidylcholine (DMPC) and polyoxyethylene surfactants demonstrated vitro cytotoxicity and in vivo efficacy in liver metastasis models by inducing apoptosis in CRC cells [89]. Thermosensitive liposomes (TSLs) co-loaded with anticancer drugs and MRI contrast agents have enabled image-guided drug release in response to mild hyperthermia, improving spatial and temporal control of chemotherapy in orthotopic CRC models [90]. Functionalization of pH-sensitive liposomes with ligands such as fibronectin-mimetic peptides or integrin α5β1-binding moieties has further enhanced selective binding and internalization by CRC cells, particularly in acidic tumor microenvironments [91]. The co-delivery of chemotherapeutics and radiotherapeutics using dual-functional liposomes has also shown synergistic effects. For instance, PEGylated liposomes co-loaded with vinorelbine, and indium-111 provided combined chemoradiotherapy, resulting in improved tumor regression compared to monotherapies [92]. Additional studies utilizing photosensitizer-conjugated liposomes (e.g., pyro pheophorbide-a and tetrapyrrole derivatives) have reported enhanced photodynamic therapy efficacy through increased intracellular accumulation and phototoxicity in HCT-116 CRC cells. Targeted delivery strategies have been explored using transferrin receptor (TfR)-binding ligands, neurotensin peptides, and tumor-specific integrins [93]. These modifications have improved the uptake of liposomes by CRC cells and increased cytotoxicity. Neurotensin-functionalized liposomes delivering irinotecan or oxaliplatin have shown a four-fold increase in efficacy in vitro, underscoring their potential in metastatic CRC therapy [94]. Genetic payloads such as plasmid DNA, shRNA, and mRNA have also been successfully incorporated into cationic liposomes. For example, liposomes delivering endostatin or tumor necrosis factor-related apoptosis-inducing ligand (TRAIL) genes have significantly reduced tumor burden and angiogenesis in xenograft models. PEGylated liposomes encapsulating shRNA targeting the kitenin gene have shown promise in inhibiting CRC progression by modulating apoptosis-related pathways [95]. Additionally, liposomes co-delivering cytosine deaminase and 5-fluorocytosine, in combination with radiotherapy, achieved over 80% tumor volume reduction in murine CRC models, with enhanced therapeutic efficacy when combined with Par-4 plasmid to potentiate 5-fluorouracil-induced apoptosis. Several liposomal formulations are currently under clinical investigation [96]. Liu et al. demonstrate that liposomal co-delivery of IOX1, a histone demethylase inhibitor, with doxorubicin synergistically enhances antitumor efficacy by downregulating PD-L1, overcoming drug resistance, and inducing immunogenic cell death. This approach promotes dendritic cell maturation, restores T-cell activity, and achieves durable tumor eradication with long-term immunological memory in murine models, offering a promising antibody-independent chemo-immunotherapy strategy [97]. Notably, Doxil^®^ (liposomal doxorubicin) and Marqibo^®^ (liposomal vincristine) have been repurposed for CRC therapy, while ThermoDox^®^, a heat-triggered doxorubicin formulation, has demonstrated a 25-fold increase in intratumoral drug concentration under localized hyperthermia, offering a promising strategy for precision chemotherapy [98].

While significant progress has been made, challenges such as tumor heterogeneity, endosomal escape, and immunosuppressive tumor microenvironments continue to limit clinical translation. Nonetheless, the convergence of liposomal nanotechnology with targeted therapy, immunomodulation, and theranostic approaches holds great promise for the next generation of CRC treatments.

## 5. Liposome-Based Drug Delivery Systems for the Treatment of Liver Cancer

According to the NIH/NCI Surveillance, Epidemiology, and End Results (SEER) Program Cancer Stat Facts for liver and intrahepatic bile duct cancer, an estimated 42,240 new cases are expected in 2025, representing 2.1% of all new cancer cases. The disease is projected to cause 30,090 deaths, accounting for 4.9% of all cancer-related deaths. The 5-year relative survival rate for cases diagnosed between 2015 and 2021 is 22.0% [99]. Primary liver cancer is frequently associated with chronic liver diseases such as hepatitis B and C infections, alcoholic liver cirrhosis, and non-alcoholic steatohepatitis (NASH). These conditions induce genetic and epigenetic alterations that activate oncogenic pathways, including RAS/RAF/MEK/ERK and PI3K/AKT, which promote tumor cell survival, proliferation, angiogenesis, and immune evasion. While liver transplantation and surgical resection remain the mainstay of treatment in early-stage HCC, advanced disease is often refractory to systemic chemotherapies due to multidrug resistance and poor drug bioavailability [100]. In this context, liposome-based nanocarriers offer a promising strategy to improve therapeutic efficacy through targeted delivery, enhanced tumor accumulation, and reduced systemic toxicity.

Thermosensitive liposomes (TSLs) have shown considerable promise in HCC therapy. For instance, doxorubicin-loaded TSLs (ThermoDox^®^), when combined with radiofrequency ablation (RFA), have demonstrated enhanced intratumoral drug accumulation and antiangiogenic effects, resulting in improved tumor suppression in preclinical xenograft models [101]. Additionally, siRNA-loaded liposomes targeting anti-apoptotic genes such as Bcl-2 have been shown to significantly reduce tumor growth in murine models, particularly when formulated with ionizable cationic lipids and phospholipids to facilitate efficient endosomal escape [102]. Targeted liposomal delivery systems have also been engineered to exploit overexpressed surface markers in HCC. Wang et al. reported the use of CD147-targeted doxorubicin-loaded liposomes, which exhibited selective cytotoxicity in Huh-7, HepG2, and HCC3736 cells by leveraging receptor-mediated endocytosis [103]. Similarly, Quagliariello et al. demonstrated that chitosan-coated liposomes encapsulating butyric acid achieved higher cytotoxicity in HepG2 cells compared to uncoated liposomes or free drug, suggesting the benefit of polymer-lipid hybrid systems [104]. In another notable study formulated sorafenib-loaded liposomes that exhibited improved pharmacokinetics, biocompatibility, and tumor regression in Hep3B xenograft mouse models, compared to free sorafenib [105]. Jiang et al. developed glycyrrhetinic acid (GA)-modified liposomes co-delivering curcumin and combretastatin A4 phosphate (CA4P), which demonstrated enhanced cellular uptake, cytotoxicity, and real-time tumor imaging via near-infrared (NIR) fluorescence in BEL-7402 HCC models [106]. Advanced targeting strategies have further enhanced the precision of liposomal delivery. For example, Longmuir et al. designed liposomes conjugated with glycosaminoglycan-binding peptides to selectively deliver doxorubicin to hepatocytes by targeting heparan sulfate proteoglycans [107]. Opanasopit et al. developed mannosylated liposomes to target Kupffer cells through mannose receptor recognition, effectively delivering immunomodulatory muramyl dipeptides and reducing metastatic burden in liver tumor-bearing mice [108]. Asialoglycoprotein receptors (ASGPR) and CD44 are also frequently overexpressed in HCC and have been used to guide liposomal targeting. Ligands such as lactosylceramide and asialofetuin have been conjugated to liposomes to facilitate receptor-mediated endocytosis and selective hepatic delivery [109]. Moreover, anti-VEGF antibody-conjugated long-circulating liposomes encapsulating sorafenib have shown potent antiangiogenic and antitumor effects in orthotopic liver tumor models [110].

Despite the encouraging preclinical and early clinical progress, no liposomal formulation has yet received regulatory approval specifically for liver cancer treatment. However, ongoing clinical trials are evaluating the safety and efficacy of formulations like ThermoDox^®^ and other targeted liposomes. Key challenges, including tumor heterogeneity, fibrosis-induced delivery barriers, and immune suppression within the tumor microenvironment, must be addressed to fully realize the clinical potential of liposome-based therapies in HCC.

## 6. Liposome-Based Drug Delivery Systems for the Treatment of Breast Cancer

According to the NIH/NCI Surveillance, Epidemiology, and End Results (SEER) Program Cancer Stat Facts for female breast cancer, an estimated 316,950 new cases are expected in 2025, comprising 15.5% of all new cancer diagnoses. The disease is projected to cause 42,170 deaths, accounting for 6.8% of all cancer-related deaths. The 5-year relative survival rate for cases diagnosed between 2015 and 2021 is 91.7% [111]. Among its various subtypes, triple-negative breast cancer (TNBC) is particularly challenging due to the absence of estrogen receptor (ER), progesterone receptor (PR), and human epidermal growth factor receptor 2 (HER2), which collectively limit the effectiveness of receptor-targeted therapies and contribute to aggressive progression and high metastatic potential [112].

Multiple genetic, hormonal, and environmental risk factors contribute to breast cancer development, including BRCA1/2 and TP53 mutations, prolonged estrogen exposure, radiation, obesity, and sedentary lifestyle. Given the molecular heterogeneity of breast tumors and limitations of conventional therapies, liposome-based drug delivery systems have emerged as a promising strategy to improve therapeutic index, reduce systemic toxicity, and overcome resistance mechanisms Targeted immunoliposomes have shown promise in preclinical and clinical settings. For instance, anti-EGFR antibody fragment (Fab) conjugated liposomes encapsulating doxorubicin or vinorelbine have demonstrated enhanced tumor-specific cytotoxicity in EGFR- and EGFRvIII-overexpressing breast cancer models, including MDA-MB-468 xenografts. Similarly, HER2-targeted immunoliposomes delivering doxorubicin have demonstrated selective uptake in HER2-positive breast cancer cells with minimal cardiotoxicity [113]. Moreover, anti-transferrin receptor (TfR)-conjugated liposomes carrying siRNA or chemotherapeutic payloads have shown increased internalization and silencing efficacy in TfR-overexpressing breast cancer cells [114]. In ER-positive tumors, estrone-conjugated liposomes have been utilized to improve receptor-mediated targeting compared to estradiol-based systems, offering higher binding affinity and reduced systemic off-target effects. For metastatic breast cancer, stealth liposomes modified with tumor metastasis-targeting (TMT) peptides and encapsulating doxorubicin have demonstrated enhanced accumulation in MDA-MB-231 and SP90, a tumor-targeting peptide identified for breast cancer, has been successfully used for conjugation to liposomes containing doxorubicin or quantum dots. These multifunctional systems offer both therapeutic and imaging capabilities, enabling targeted delivery, enhanced apoptosis, anti-angiogenesis, and real-time fluorescence tracking of tumor burden [115]. Similarly, the vasoactive intestinal peptide receptor (VIP-R), highly expressed in certain breast cancers, has been exploited for targeted delivery of radiolabeled liposomes, allowing non-invasive tumor imaging and potential theranostic applications [116]. Recent innovations have also incorporated pH-sensitive liposomes for controlled intracellular drug release. Juliana de Oliveira Silva et al. developed folic acid-conjugated, long-circulating liposomes encapsulating doxorubicin that exhibited enhanced cellular uptake and antitumor activity in 4T1 breast tumor-bearing mice, especially under acidic tumor microenvironment conditions [117].

In terms of regulatory progress, several liposomal formulations have been approved for clinical use in breast cancer therapy. Doxil^®^, Lipodox^®^, and Myocet^®^—all doxorubicin-encapsulated liposomal formulations—have demonstrated improved safety profiles, particularly with reduced cardiotoxicity, and are currently used in the treatment of metastatic breast cancer and recurrent cases. In addition, numerous investigational liposomal agents are in preclinical and clinical development, targeting TNBC and HER2-positive tumors using advanced surface modifications and co-delivery strategies.

## 7. Liposome-Based Drug Delivery Systems for the Treatment of Ovarian Cancer

According to the NIH/NCI Surveillance, Epidemiology, and End Results (SEER) Program Cancer Stat Facts for ovarian cancer, an estimated 20,890 new cases are expected in 2025, accounting for 1.0% of all new cancer diagnoses. Ovarian cancer is projected to cause 12,730 deaths, representing 2.1% of all cancer-related deaths. The 5-year relative survival rate for cases diagnosed between 2015 and 2021 is 51.6% [118]. Metastasis in ovarian cancer predominantly occurs via peritoneal dissemination, as opposed to lymphatic or hematogenous routes. This unique dissemination pattern poses significant challenges for conventional therapeutic strategies such as cytoreductive surgery, systemic chemotherapy, and radiotherapy. Effective management of peritoneal metastases requires innovative approaches to target disseminated tumor cells, minimize recurrence, and overcome drug resistance [119].

One of the key factors influencing the aggressiveness and prognosis of ovarian cancer includes the expression of epithelial–mesenchymal transition (EMT) markers, which facilitate tumor invasion and metastatic spread. Additionally, the presence of cancer stem cell populations and chemoresistance mechanisms, particularly resistance to platinum-based therapies, further complicates treatment [120]. Recent advancements in nanotechnology have led to the development of liposomal drug delivery systems (LNPs) that improve pharmacokinetics and target tumor-specific markers. One notable innovation involves CD24-targeted liposomes loaded with cisplatin and fluorescent marker Cy5.5, developed by Ashihara et al. [121]. CD24, overexpressed in approximately 70% of ovarian cancer cases, plays a critical role in promoting EMT, drug resistance, and tumor progression via activation of the Akt and ERK pathways [122]. In CaOV-3 xenograft models, the targeted liposomes demonstrated substantial tumor suppression and EMT reversal. Mechanistically, the liposomes inhibited Snail, a transcriptional repressor of E-cadherin, thereby restoring E-cadherin levels and impeding peritoneal dissemination. Theranostic approaches combining imaging and treatment are being explored for ovarian cancers with HER2/ErbB2 overexpression [123]. Han et al. (2020) [124] developed liposomes conjugated with a recombinant EC1-peptide fused to Gaussia luciferase (GLuc) for real-time bioluminescent imaging. These nanocarriers were selectively internalized by HER2-positive SKOV3 cells and co-delivered therapeutic agents such as HPTS for in vivo imaging and intracellular targeting, showing promise for precision diagnostics and therapy. Additional innovations include RGD-modified liposomes for targeted delivery of gemcitabine, a nucleoside analog chemotherapy. These liposomes demonstrate enhanced cellular uptake and pro-apoptotic effects in SKOV3 cells [125]. In another study, EphA2-targeted siRNA delivered via liposomal nanoparticles and co-loaded with paclitaxel showed potent anti-tumor activity when administered intraperitoneally in orthotopic ovarian tumor models, resulting in tumor suppression ranging from 48% to 81% [126]. Targeting Focal Adhesion Kinase (FAK) using neutral DOPC-based siRNA liposomes has also shown promise in multiple ovarian cancer models, including chemo resistant A2780-CP20 cells [127]. FAK-siRNA-DOPC reduced tumor weights by up to 72%, while combination therapy with docetaxel inhibited angiogenesis, VEGF expression, and matrix metalloproteinase-9, leading to increased apoptosis of both tumor and endothelial cells. Incorporation of ceramides into liposomal membranes has been found to enhance membrane permeability and induce apoptosis, particularly when conjugated with transferring for improved targeting. These formulations exhibited therapeutic efficacy in HeLa cells (in vitro) and A2780 xenograft mouse models (in vivo), showing increased sensitivity to chemotherapeutics [128]. 

Among liposomal formulations, Lipodox^®^, a pegylated liposomal doxorubicin formulation, has received FDA approval for the treatment of ovarian cancer, demonstrating improved circulation time and reduced cardiotoxicity. Several other liposomal candidates are currently in clinical trials, focusing on both chemotherapeutic delivery and gene silencing strategies.

## 8. Liposome-Based Drug Delivery Systems for Brain Tumors and Neuroblastoma

Epidemiological Perspective: According to the NIH/NCI Surveillance, Epidemiology, and End Results (SEER) Program, brain and other nervous system cancers are expected to account for 24,820 new cases and 18,330 deaths in 2025, representing 1.2% of all new cancer diagnoses and 3.0% of cancer-related deaths, respectively. The 5-year relative survival rate remains low at 33.0% for cases diagnosed between 2015 and 2021 [129]. The therapeutic challenge is compounded by the restrictive nature of the blood–brain barrier (BBB) and blood–brain tumor barrier (BBTB), which severely limit drug penetration. Moreover, the enhanced permeability and retention (EPR) effect, a key mechanism in peripheral tumors, is often underexpressed in brain neoplasms. Thus, there is a critical need for nanocarrier systems capable of overcoming these barriers to improve therapeutic outcomes [130]. Liposomal Strategies in Brain Tumors: Liposomal nanoparticles (LNPs) offer a compelling platform due to their ability to encapsulate both hydrophilic and hydrophobic agents, protect drugs from degradation, and facilitate active transport across the BBB [131]. Advanced liposomes employ targeting mechanisms such as receptor-mediated transcytosis (RMT), transporter-mediated transcytosis, and adsorptive-mediated transcytosis (AMT). For example, dual-targeting immunoliposomes loaded with temozolomide and conjugated to Angiopep-2 for glioblastoma stem-like cells, demonstrating potent cytotoxicity and tumor inhibition in intracranial xenograft models [132]. Similarly, Zhao et al. developed pH-responsive liposomes conjugated with the glioma-targeting peptide H7K(R2)2, which selectively released doxorubicin in acidic tumor microenvironments, significantly prolonging survival in orthotopic glioma models [133].

Multifunctional liposomes have also been designed for theranostics. Thermosensitive formulations with Gd-DOTA or Gd-DTPA-BSA enable MRI-guided, ultrasound-triggered release, while fluorescent dye-labeled liposomes improve intraoperative glioma margin visualization [134]. Targeting IL-4R overexpressed in gliomas using AP1 peptide-conjugated lipoplatin has shown enhanced cisplatin delivery and tumor suppression in orthotopic GBM models [135]. Radioisotope-loaded liposomes are being investigated for brachytherapy, further expanding the therapeutic utility of these nanocarriers. Liposomal Therapy in Neuroblastoma: Neuroblastoma (NB), the most common extracranial solid tumor in children, exhibits marked biological heterogeneity ranging from spontaneous regression to aggressive, therapy-resistant forms [136]. The toxicity of systemic chemotherapy in pediatric patients necessitates targeted approaches like liposomal delivery to minimize off-target effects. Targeting survivin, overexpressed in high-risk NB, Gholizadeh et al. developed anti-GD2 immunoliposomes encapsulating YM155, achieving selective uptake and significant tumor inhibition in preclinical models [137]. For gene silencing, GD2-Fab-conjugated cationic liposomes delivering ALK-targeted siRNA effectively suppressed tumor growth and angiogenesis, extending survival in xenograft models [138]. Nevertheless, several candidates are progressing through advanced preclinical and early clinical stages.

LNP-based drug delivery systems have demonstrated exceptional versatility in addressing the therapeutic challenges of brain tumors and neuroblastoma. With their potential for precise targeting, multifunctional integration, and reduced systemic toxicity, liposomes represent a transformative advancement in nanomedicine. Continued innovation in ligand engineering, stimuli-responsive release, and imaging-guided theranostics are expected to accelerate their clinical translation in neuro-oncology.

## 9. Liposome-Based Drug Delivery Systems for the Treatment of Pancreatic Cancer 

Pancreatic cancer, predominantly in the form of pancreatic ductal adenocarcinoma (PDAC), remains among the most lethal malignancies globally. According to the NIH/NCI Surveillance, Epidemiology, and End Results (SEER) Program Cancer Stat Facts for pancreatic cancer, an estimated 67,440 new cases are expected in 2025, representing 3.3% of all new cancer diagnoses. Pancreatic cancer is projected to cause 51,980 deaths, accounting for 8.4% of all cancer-related deaths. The 5-year relative survival rate for cases diagnosed between 2015 and 2021 is 13.3% [139]. 

The molecular pathology of PDAC involves complex signaling cascades, including KRAS mutations, PI3K/Akt, and Hedgehog (Hh) pathways, which contribute to tumor initiation, angiogenesis, desmoplasia, and therapy resistance [140]. These features, coupled with the dense stromal barrier, present significant challenges for drug delivery, making liposomal nanocarriers a promising platform for therapeutic innovation. To improve drug specificity and diagnostic imaging, ultra-small superparamagnetic iron oxide nanoparticles (USPIOs) have been incorporated into liposomes conjugated with anti-mesothelin (MSLN) monoclonal antibodies. These liposomes, co-loaded with doxorubicin, have demonstrated enhanced accumulation and tumor growth inhibition in pancreatic tumor xenograft models through MRI-guided imaging and targeted therapy [141]. LNPs carrying shRNA sequences targeting PDX-1 and ZIP4, two oncogenic regulators in pancreatic cancer, have been evaluated in immunodeficient mouse models, where they significantly delayed tumor progression and reduced tumor burden [142]. Gemcitabine, a nucleoside analog and frontline chemotherapeutic for PDAC, suffers from low membrane permeability and rapid systemic clearance. To overcome this, thermosensitive liposomes (TSLs) have been engineered to co-deliver gemcitabine under mild hyperthermic conditions (≈42 °C). In MiaPaCa-2 xenograft models, these TSLs enhanced intratumorally accumulation of gemcitabine by 3.5-fold compared to the free drug and significantly inhibited tumor progression [143]. However, although gadolinium (Gd)-loaded TSLs enhanced drug accumulation, they failed to improve MRI signal contrast in ex vivo evaluations, indicating the need for further optimization of contrast agent loading and release kinetics [144]. Theranostic liposomes tagged with syndecan-1 (Sdc1) have been developed to target insulin-like growth factor 1 receptor (IGF1R)-overexpressing pancreatic tumors. These particles encapsulate near-infrared dyes and are visualized using multispectral optoacoustic tomography (MSOT). The Sdc1-IGF1R-α5β3 integrin axis provides target specificity, reducing off-target effects and enhancing resolution in orthotopic mouse models [145]. Rachamala et al. demonstrated that dual targeting of Syndecan-1 (SDC1) and Glucose Transporter-1 (GLUT1) using a novel lipid-based delivery system significantly enhances therapeutic efficacy and overcomes chemoresistance in pancreatic ductal adenocarcinoma (PDAC). By co-delivering therapeutic agents to two critical regulators of tumor progression and metabolic adaptation, this approach led to improved cellular uptake, increased apoptosis, and marked tumor growth inhibition in preclinical models. The study underscores the potential of rationally designed nanocarriers targeting multiple tumor-associated pathways to address the therapeutic challenges in refractory cancers such as PDAC. These findings pave the way for translational development of multi-targeted nanomedicine strategies for aggressive and drug-resistant malignancies [8]. Rachamala et al. demonstrated that liposomal delivery of thymoquinone markedly improves its pharmacokinetic behavior and antitumor efficacy. The liposome-encapsulated formulation exhibited enhanced systemic stability, prolonged circulation time, and increased tumor-specific accumulation, leading to superior therapeutic outcomes. Compared to free thymoquinone, the liposomal form significantly inhibited tumor growth while minimizing systemic toxicity. These findings highlight the potential of liposomal nanocarriers in optimizing the clinical utility of bioactive phytochemicals like thymoquinone for effective cancer therapy [12]. Transferrin receptor (TfR)-targeted liposomes, incorporating Gd-DTPA as a contrast agent, have shown promise in MRI-based molecular imaging of pancreatic tumors. This dual-functional system allows for both tumor visualization and potential co-delivery of therapeutic agents, facilitating real-time monitoring of treatment response [146]. To improve intratumoral penetration, liposomes have been functionalized with tumor vasculature-targeting peptides: p15-RGR peptide targets PDGFR-β on tumor-associated pericytes p46-RGD peptide binds to αvβ3 integrins on tumor endothelial cells. These modifications significantly enhance liposome accumulation in pancreatic tumors, improving drug bioavailability and therapeutic efficacy [147]. One of the most notable successes in liposomal drug development is Onivyde^®^, a liposomal irinotecan formulation approved by the FDA for metastatic pancreatic cancer. Onivyde, used in combination with fluorouracil and leucovorin, has shown superior overall survival compared to conventional irinotecan therapy, particularly in gemcitabine-refractory cases [148].

Despite such progress, most liposomal formulations for pancreatic cancer remain in preclinical or early clinical stages. Significant efforts are underway to address the heterogeneous tumor microenvironment, dense stromal barrier, and immune suppression that limit drug delivery and efficacy. Liposomal drug delivery systems have demonstrated substantial potential in improving therapeutic outcomes in pancreatic cancer. Through targeted delivery, stimuli-responsive release, and integrated imaging capabilities, liposomes address key limitations of traditional therapies. However, continued research is required to translate these innovations from preclinical models to clinical applications, particularly in overcoming stromal desmoplasia and enhancing intratumoral drug penetration.

## 10. Liposome-Based Drug Delivery Systems for the Treatment of Sarcoma

According to the NIH/NCI Surveillance, Epidemiology, and End Results (SEER) Program Cancer Stat Facts for soft tissue cancers, an estimated 13,520 new cases are expected in 2025, accounting for 0.7% of all new cancer diagnoses. These cancers are projected to cause 5410 deaths, representing 0.9% of all cancer-related deaths. The 5-year relative survival rate for cases diagnosed between 2015 and 2021 is 66.0% [149].

Despite advancements in diagnosis and surgery, the prognosis for advanced or metastatic sarcoma remains poor due to limited responsiveness to conventional chemotherapy and radiotherapy [150]. One subtype, Kaposi’s sarcoma (KS), is an Angio proliferative neoplasm of endothelial origin commonly associated with HHV-8 infection and frequently observed in immunocompromised patients, including those with HIV/AIDS or post-transplant immunosuppression [151]. This form of sarcoma is highly vascularized and relies on abnormal angiogenesis, making it a viable target for nanoparticle-based therapies. Kaposi’s sarcoma and some soft tissue sarcomas overexpress neural cell adhesion molecules (NCAMs), which have become attractive molecular targets. developed NCAM-targeted liposomes conjugated with the C3d-mimetic peptide, co-loaded with doxorubicin and gadolinium-DOTAMA, for combined chemotherapy and MRI imaging [152]. 

The first FDA-approved liposomal formulation for sarcoma treatment was Doxil^®^, a pegylated liposomal doxorubicin, approved specifically for Kaposi’s sarcoma. It has shown reduced cardiotoxicity, prolonged circulation time, improved therapeutic index compared to free doxorubicin. Despite these advances, most sarcoma-directed liposomal systems remain in preclinical or early clinical trial stages, and the challenge remains in addressing the heterogeneity of sarcoma subtypes.

## 11. Lipid Nanoparticle-Based Drug Delivery Systems for the Treatment of Leukemia 

According to the NIH/NCI Surveillance, Epidemiology, and End Results (SEER) Program Cancer Stat Facts for leukemia, approximately 66,890 new cases are projected in 2025, representing 3.3% of all new cancer diagnoses. Leukemia is expected to cause 23,540 deaths, accounting for 3.8% of all cancer-related deaths. The 5-year relative survival rate for cases diagnosed between 2015 and 2021 is 67.8% [153]. Leukemia, a group of hematopoietic malignancies, originates from the malignant transformation of hematopoietic stem cells (HSCs) or committed progenitor cells in the bone marrow or peripheral blood. It is broadly categorized into acute or chronic leukemias, and further into lymphoid or myeloid lineages, including acute lymphoblastic leukemia (ALL), acute myeloid leukemia (AML), chronic lymphocytic leukemia (CLL), and chronic myeloid leukemia (CML) [154]. The incidence, mortality, and survival outcomes of leukemia are strongly influenced by the age at diagnosis, genetic mutations, treatment accessibility, and disease subtype [155]. While advances in chemotherapy, immunotherapy, and stem cell transplantation have improved outcomes in certain subtypes, drug resistance, relapse, and systemic toxicity remain major challenges. The advent of lipid-based nanocarriers, particularly LNPs, has provided a platform for targeted delivery, gene therapy, and immune modulation in hematologic malignancies [23]. RNAi technologies have gained traction in leukemia research, with LNPs serving as ideal carriers for siRNA, shRNA, and more recently, mRNA therapies. LNPs protect RNA molecules from nuclease degradation, facilitate endosomal escape, and improve biodistribution. In acute myeloid leukemia (AML), FLT3 mutations and BCL2 overexpression are key drivers of proliferation and survival. Recent studies have developed LNPs encapsulating siRNA against FLT3-ITD mutations or BCL2, which demonstrated: effective downregulation of target genes, reduced blast proliferation, and increased apoptosis in AML cell lines and patient-derived xenograft (PDX) models [156]. For instance, in a 2022 study published in Molecular Therapy, siRNA-loaded LNPs targeting BCL2 significantly prolonged survival in NOD-SCID mice inoculated with AML blasts, showing potential as a combinatorial strategy with venetoclax. Immunoliposomes, or antibody-conjugated LNPs, offer precise targeting of leukemia cells through surface antigens such as CD33 and CD123 in AML, CD19 and CD22 in B-cell ALL, CD20 in CLL [157].

LNPs conjugated with anti-CD33 antibodies for the delivery of cytarabine (Ara-C) have demonstrated improved bone marrow selectivity, enhanced drug retention, and reduced systemic toxicity in orthotopic AML murine models. This approach is currently being evaluated in combination with immune checkpoint inhibitors to further reduce leukemic burden while preserving host immunity [158]. Emerging LNPs are now being designed to deliver CRISPR-Cas9 components to edit oncogenes or restore tumor suppressor genes in leukemia. For example: A 2023 preclinical study published in Nature Biomedical Engineering reported the use of LNPs carrying Cas9 mRNA and sgRNA targeting the TET2 gene in AML models, achieving >60% editing efficiency and reversing leukemogenesis. Such non-viral vectors offer safer alternatives to lentiviral or retroviral systems for ex vivo or in vivo gene correction [27]. While LNP technologies are extensively applied in solid tumors and vaccines (e.g., mRNA COVID-19 vaccines), their application in hematologic malignancies is now gaining momentum. Several LNPs for leukemia are in preclinical or Phase I trials, and liposomal formulations such as Vyxeos^®^ (a liposomal combination of daunorubicin and cytarabine) have already been FDA approved for therapy-related AML and AML with myelodysplasia-related changes (AML-MRC). Vyxeos prolongs drug exposure time and delivers fixed synergistic ratios, improving remission rates and overall survival [19].

Lipid nanoparticle-based drug delivery systems represent a powerful tool for advancing leukemia therapy. Through targeted delivery of RNA molecules, gene editing systems, and chemotherapeutics, LNPs overcome many limitations of systemic treatments, such as poor selectivity, immune evasion, and drug resistance. The ability to functionalize LNPs with antibodies, peptides, and ligands targeting leukemia-specific antigens paves the way for precision medicine. Despite promising preclinical and early clinical results, challenges remain, including bone marrow targeting, immune recognition, and long-term safety. Nevertheless, ongoing innovation in nanocarrier engineering, bioconjugation, and multifunctional payload delivery holds strong potential to shift LNP-based therapies for leukemia from bench to bedside.

Future directions and prospects of LNPs: The future of LNPs centers on enhancing targeting precision through biomimetic coatings and organ-specific ligands for improved tissue selectivity. AI-driven formulation will enable personalized LNP design by optimizing lipid composition and pharmacokinetics. Efforts will also focus on scalable, cost-effective manufacturing to expand accessibility. Overcoming barriers to cross-tissue delivery especially to the brain and solid tumors remains a major challenge. Next-generation LNPs will integrate with mRNA vaccines, CRISPR systems, and immunotherapies, expanding their therapeutic reach. Smart LNPs incorporating imaging agents (e.g., fluorescent dyes, MRI/PET tracers) will enable real-time tracking and theranostics, while stimulus-responsive LNPs will refine treatment timing and precision. LNPs are also being developed to modulate the TME by delivering immunomodulators or gene editors to reshape immune responses, particularly targeting macrophages, dendritic cells, and immune checkpoints. Biomimetic strategies, such as cancer cell membrane coatings, can enhance tumor selectivity and reduce off-target effects. Integration with wearable or implantable devices will allow localized, responsive delivery based on physiological cues (e.g., pH, temperature), with real-time feedback for dynamic treatment adjustments. Clinically, successful translation will require rigorous preclinical testing for stability, toxicity, and biodistribution; scalable GMP-compliant manufacturing; and robust clinical trials demonstrating efficacy and safety. Post-market surveillance will ensure long-term performance. AI and advanced formulation platforms will streamline development and regulatory processes, accelerating the impact of LNPs in precision medicine.

## 12. Conclusions

Nanomedicine, particularly through the development and application of lipid LNPs and LNPs, stands as a pivotal advancement in the ongoing battle against cancer. This review highlighted the remarkable versatility of these nanoscale platforms in addressing the fundamental limitations of conventional oncology treatments, such as systemic toxicity, poor drug solubility, and lack of tumor specificity. By enabling targeted delivery of a diverse array of therapeutic payloads ranging from traditional chemotherapeutics and potent RNA-based agents (siRNA, mRNA) to sophisticated gene editing tools (CRISPR) and immunomodulators, nanocarriers offer unprecedented potential for enhancing treatment efficacy while minimizing harm to healthy tissues. The clinical success of LNP-based mRNA vaccines has served as a powerful validation of this technology, catalyzing renewed enthusiasm and investment in its application across various therapeutic areas, including oncology. Similarly, established liposomal formulations like Doxil^®^, Marqibo^®^, and Onivyde^®^ demonstrate the tangible clinical benefits of nanocarrier-mediated drug delivery in specific cancer types, even as extensive preclinical research continues to explore liposomal applications across a broader spectrum of malignancies, including melanoma, lung, colorectal, liver, breast, ovarian, brain, pancreatic, sarcoma, neuroblastoma, and leukemia. Despite these significant strides, the path from bench to bedside remains complex. Challenges related to large-scale manufacturing, ensuring consistent quality, achieving precise biodistribution, overcoming biological barriers like the BBB, managing potential immunogenicity, and navigating regulatory hurdles persist. However, the field is rapidly evolving. Innovations such as next-generation ionizable lipids, biomimetic coatings, sophisticated targeting strategies (including SORT technology), and the integration of artificial intelligence in formulation design are paving the way for smarter, safer, and more effective nanomedicines. Looking ahead, the convergence of nanotechnology with immunotherapy, personalized medicine (e.g., neoantigen vaccines), and theranostics promises to usher in a new era of cancer care. The continued refinement of LNP and liposome platforms, driven by interdisciplinary collaboration and technological breakthroughs, holds immense promise for transforming cancer treatment paradigms, improving patient outcomes, and ultimately realizing the vision of truly personalized and precise oncology.

## Figures and Tables

**Table 1:** AI-assisted advances in LNP formulation design (2020–2025).

Aim	AI/ML Method	Key Outcome	Relevance
Predict ionizable lipid properties and mRNA delivery	ML predictive models	Identified novel lipids with improved potency	Accelerates lipid library screening
Inverse design of LNP formulations	Transformer models	Predicted formulations with high transfection	Automates formulation discovery
Optimize microfluidic LNP manufacturing	XGBoost + Bayesian optimization	Improved size, EE%, potency reproducibility	Enhances scalability and CQA control
Predict biodistribution of NPs	QSAR + ML interpretability	Accurately forecasted organ/tumor uptake	Guides precision oncology delivery
Automated morphology analysis	Computer vision (LNP-MOD)	Linked cryo-EM features to efficacy	Enables theranostic optimization

**Table 2:** Clinical Trials Landscape (2020–2025) of LNPs.

Application Area	Example/Trial	Sponsor/Developer	Clinical Focus	Status/Impact
Infectious Diseases (COVID-19)	mRNA-1273 (Moderna) BNT162b2 (Pfizer/BioNTech)	Moderna; Pfizer/BioNTech	LNP-based mRNA vaccines against COVID-19	Emergency/global approval; landmark success
Cancer Vaccines	BNT122 (BioNTech/Genentech) mRNA-4157 (Moderna/Merck)	BioNTech/Genentech; Moderna/Merck	Personalized mRNA neoantigen vaccines for melanoma and solid tumors	Phase I/II trials; promising immune responses
Gene Editing	NTLA-2001 (Intellia)	Intellia Therapeutics	In vivo LNP delivery of CRISPR-Cas9 for transthyretin amyloidosis (ATTR)	First-in-human CRISPR therapy; early clinical success
RNAi Therapies	Patisiran (ONPATTRO)	Alnylam Pharmaceuticals	siRNA delivery for hereditary transthyretin amyloidosis	FDA/EMA approved; expanded use 2020–2025
Cancer Immunotherapy/CAR-T	LNP-delivered immunomodulatory mRNA (preclinical to early trials)	Multiple (BioNTech, Moderna, academic consortia)	Enhancing CAR-T and immune checkpoint therapies	Early-phase, strong preclinical support
Autoimmune and Rare Diseases	Various pipeline candidates	Multiple developers	LNPs for autoimmune disorders, liver-targeted gene therapy	Ongoing early clinical trials

## Data Availability

No new data were created or analyzed in this study.
